# First results of the Strengths and Difficulties Questionnaire, applied as a screening tool for psychosocial difficulties in pediatric audiology

**DOI:** 10.1007/s00405-023-07979-x

**Published:** 2023-04-21

**Authors:** Tjeerd J. de Jong, Marc P. van der Schroeff, Marieke D. Achterkamp, Jantien L. Vroegop

**Affiliations:** 1 Department of Otorhinolaryngology, Head and Neck Surgery, Erasmus University Medical Center, PO box 2040, 3000 CA Rotterdam, The Netherlands; 2 Department of Child and Adolescent Psychiatry/Psychology, Erasmus University Medical Center, Rotterdam, The Netherlands

**Keywords:** Children, Hearing loss, Psychosocial difficulties, Strengths and Difficulties Questionnaire (SDQ), Psychological assessment, Speech perception

## Abstract

**Purpose:**

Despite major improvements in rehabilitation possibilities, children with sensorineural hearing loss are at risk for psychosocial difficulties. These difficulties can impact their educational and career achievements and may be two to three times more common in children with hearing loss compared to those with normal hearing. Early identification of psychosocial difficulties can be facilitated using the Strengths and Difficulties Questionnaire (SDQ) and may improve outcomes and quality of life. We implemented the SDQ into the clinical follow-up of children with hearing loss in a tertiary referral hospital. With this, prevalence and severity of difficulties in specific psychosocial domains and several predictors were investigated.

**Methods:**

A retrospective, cross-sectional investigation was performed of the following factors in association with the SDQ results: type of hearing device, type and degree of hearing loss, speech perception in quiet and in noise, and type of schooling.

**Results:**

Between June 2020 and January 2022, parents of 312 children (age 4–18) completed the SDQ. An additional 113 child-reports were completed. The response rate of the parents was 69%. Problems with peer relationships and prosocial behavior were the most affected areas with clinically elevated scores in 22% of the children. Psychosocial difficulties were distributed similarly across types of hearing device, nature and degrees of hearing loss, and educational settings. Better speech perception in quiet was significantly associated with fewer parent-reported conduct problems.

**Conclusion:**

The results of the present study suggest that children with hearing loss may be at risk of experiencing challenges with social interactions and attachment in social contexts. Using the SDQ in clinical follow-up may improve the chances for early psychological assessment and intervention. In addition, the study found that children’s mental health may be impacted by their communication abilities.

**Supplementary Information:**

The online version contains supplementary material available at 10.1007/s00405-023-07979-x.

## Introduction

Despite major improvements in rehabilitation possibilities and the expanse of modern technology, children with hearing loss (HL) are at risk for psychosocial difficulties [1]. The children’s risk for poorer receptive and expressive language [2–4], and possible delays in cognitive development and executive functioning [5, 6] may impact their psychosocial functioning such as social behavior, peer relationships [7–9], and educational and career achievements [10]. The majority of papers on this subject report a 20–40% prevalence rate of psychosocial disadvantages [1]. In comparison with normal hearing (NH) peers, the risk for experiencing one or more problems is two times higher in children with HL [8, 11–13], and even odds ratios up to 3.9 for problems in socio-emotional domains are reported [14].

The high prevalence of psychosocial difficulties among children with HL suggests that current interventions and services may not be sufficient to meet the children’s needs and address the challenges they face. This highlights the need for more comprehensive and holistic approaches, addressing their social and emotional needs in addition to adequate rehabilitation. The psychosocial wellbeing of these children has a significant impact on their quality of life [15] and ability to understand and cope with their condition as well as their success in rehabilitation programs. Also, earlier support initiation is associated with better psychosocial outcomes [16, 17]. Therefore, early identification could benefit patients and reduce healthcare costs [18].

Early identification of psychosocial difficulties can be facilitated with the use of appropriate screening tools. One questionnaire that has been extensively used in healthcare is the Strengths and Difficulties Questionnaire (SDQ), developed by Goodman in 1997. The SDQ has been validated as a multi-informant (both parents and children) screening tool for psychosocial problems in populations of children and adolescents, and normative data are available for several countries, including The Netherlands [19]. It has been used in studies on children with HL [1], and found to be effective in identifying their psychosocial needs [20].

Several factors, including type of hearing device [21], degree of HL [22], and speech perception abilities in quiet and noise [12, 23] have been found to predict psychosocial outcomes. Poor speech perception abilities can result in social isolation [24], and the type of education a child receives can impact their ability to communicate in group settings and develop social skills [25].

To early identify at-risk children, as of June 2020, our audiology department implemented the SDQ in the clinical follow-up of these children. Using the SDQ, we have been able to gain a better understanding of the children’s overall wellbeing and identify potential predictors that may be affecting their social and emotional development.

The present article (1) reports on these findings regarding the prevalence, severity, and specific domains of psychosocial difficulties among children with HL, using the parent- and child administered SDQs, and comparing the results to data on Dutch peers with NH; (2) aims to identify any correlations that may exist between factors such as type of hearing device, degree of HL, speech perception abilities in quiet and in noise, type of education and psychosocial difficulties.

## Materials and methods

### Strengths and Difficulties Questionnaire

The Strengths and Difficulties Questionnaire (SDQ) is a frequently used short screening form on psychosocial difficulties in children. It is validated for seeking psychosocial problems, strengths of the child and impact of psychosocial problems on daily functioning, in both clinical and research samples [20, 26, 27]. It consists of 25 items, grouped into 5 scales (emotional problems, conduct problems, hyperactivity and inattention, peer problems, and prosocial behavior) [28]. The former 4 scales assess difficulties, the latter one assesses strengths. Scores are divided into ‘close to average’ (80% of the population), ‘slightly raised’ (10%), ‘high’ (5%), and ‘very high’ (5%) [19]. We used the Dutch parent version for SDQ reports on children between 4 and 18 years of age, and the Dutch child version for children between 11 and 18 years of age [29]. The study of Maurice-Stam et al. (2018) provided contemporary parent-rated SDQ scores of Dutch normal hearing children between 4 and 18 years. Norm scores were reported as the percentage of children scoring in the clinical range (high or very high) in the age categories: 4–5 years, 6–11 years, and 12–18 years. We used this as benchmark for comparison with our sample.

### The clinical implementation of the SDQ

**Fig. 1:**
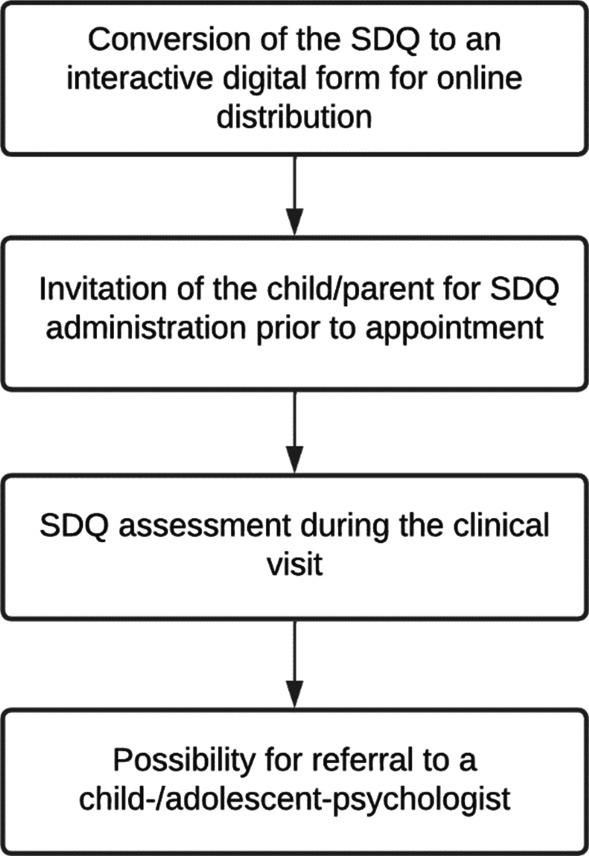
Flowchart of the SDQ’s implementation in the clinical follow-up of children with hearing loss. SDQ is an abbreviation for Strengths and Difficulties Questionnaire

**Fig. 2:**
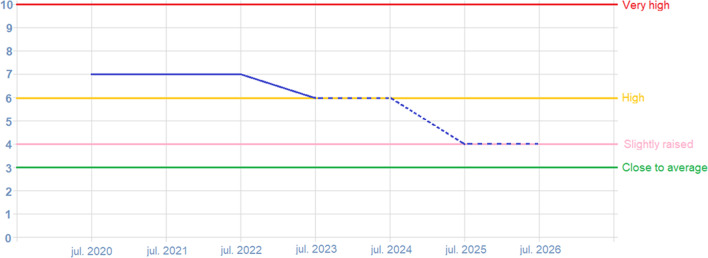
SDQ emotional problems of a fictitious patient over time. The SDQ score of the emotional problems scale as displayed in the clinician’s online dashboard. The blue line represents the score, plotted against time. A score over 6 is deemed ‘very high’, over 4 ‘high’, over 3 ‘slightly raised’, and otherwise ‘close to average’. In this fictitious patient, the SDQ emotional problems score decreases over time

A stepwise approach was followed for the clinical implementation of the SDQ into the clinical care of the audiology department in Erasmus University and Medical Center - Sophia Children’s Hospital in Rotterdam. See Fig. 1 for the steps:

1. The Dutch child- and parent versions of the SDQ [29] were converted to a digital version (as shown in Supplementary Fig. 1). Scores were automatically computed for the different scales. Scores would not be shown to the informants, but were available to the involved clinicians and researchers. For the distribution of the SDQ’s, we used Limesurvey [30]. This is an online platform, through which questionnaires can be designed, distributed, administered and assessed. It has been used for several years in the Erasmus University and Medical Center, where it is implemented as Healthcare Monitor [31]. All children who were fitted with either a hearing aid (HA; including bone conducting devices) or cochlear implant (CI) received the SDQ prior to their annual clinical appointment.
2. Forms were available for administration 10 days before the children’s clinical appointment with their audiologist, and 7 days after. Parents received an email when the SDQ was available for administration, containing a uniform resource locator (URL) to the SDQ linked to their child’s patient file, and an enclosed information letter.
3. During the clinical visit, the scores were available for assessment by the audiologist or speech therapist through a dashboard with the SDQ report on the different scales (Fig. 2). In case of elevated scores, a child-/adolescent-psychologist was consulted for their interpretation of the results with respect to the child’s (psychological) background. Subsequently, elevated scores were discussed with the child and their parents. This allowed the child and their parents to bring about topics that would otherwise have been left unconsidered.
4. When the child and/or their parents let on willingness for further review of the possible psychosocial difficulties, they could be referred for consultation on short notice by a psychologist in the (same) audiology department.

### Study population

Since June 2020, all parents of children from the age of 4 up to and including the age of 18 were asked to participate (parent-report), as well as the children themselves (child-report), from the age of 11 up to and including the age of 18. Scores of the SDQs were gathered of children fitted with CIs and/or HAs, between June 2020 and January 2022. Children were included with either 1 parent-report, or both 1 parent- and 1 child-report. Through patient-file assessment, the following baseline characteristics were gathered of all children: age at diagnosis (i.e., first consultation in an audiology department); age at device (i.e., first prescription of hearing aids or first implantation); presence of additional needs (i.e., co-occurring conditions unrelated to the children’s hearing loss); type of education (as reported by the parents).

### Hearing loss assessment

Hearing thresholds in children fitted with HAs were measured with pure-tone audiometry, administered during their clinical appointment. This was done with a clinical audiometer, calibrated according to ISO standard 389-1. Hearing thresholds were determined by the sound pressure in decibels hearing level (dB HL) at which a pure tone was audible in 2 out of 3 ascents, this is in accord with the shortened ascending method based on ISO standard 8253-1. We calculated the pure-tone air conduction average (PTA4) over the frequencies of 500, 1000, 2000, and 4000 Hz (Hz) of the best hearing ear. If an air–bone gap of ≥ 10 dB HL was present, the hearing loss was classified as conductive in nature. Otherwise, it was classified as sensorineural or “perceptive” hearing loss.

### Speech perception assessment

Speech perception in quiet was measured for both CI users and HA users with the NVA-lists [32]. The NVA-lists consist of 12 monosyllabic words, of which 11 are included in the total score, and 1 is used for practice. All the words are balanced in phonemes (consonant–vowel–consonant). This resulted in 33 phonemes per list to be included in the total score. The word lists were presented at 45 dB SPL (decibels sound pressure level) for the best aided condition. Ceiling effects occurred when lists were presented at 65 dB SPL, where children would often score 95–100%, therefore, results at 65 dB SPL were left out.

For children with a score of at least 50% at 65 dB SPL, the children’s speech perception abilities in noise were measured with the Digits in Noise (DIN) test [33]. The test contains triplets of individual digits [e.g. “nul-vier-twee” (zero-four-two)], spoken by a male speaker. Utterances are played out to a background of speech spectrum masking noise, which was kept at 65 dB SPL. The children’s speech reception thresholds (SRTs) were calculated as the average signal to noise ratios (SNR) of trials 5 to 25. Whenever available, the SRTs were calculated with the second list administered during the DIN test, otherwise, the SRT of the first list was used. The DIN test was performed in the children’s best aided condition. Due to time constraints, not all children performed the DIN test during their clinical appointment.

### Statistics

Comparisons of population demographics were performed with one-way ANOVA (analysis of variance) models and the *χ*^2^ test. For the analyses of SDQ results, nonparametric tests were used because of the non-normality of the scores (i.e., positive skew). The Wilcoxon’s paired-samples nonparametric *t* test was used to investigate differences between parent- and child-reports. To test differences between two groups, the Mann–Whitney *U*’s independent-sample nonparametric t-test was used. Differences between more than two groups were investigated with the Kruskal Wallis’ one-way analysis of variance. Correlation analyses were performed with one- and two-sided Spearman tests. We used the Benjamini–Hochberg method to control the false discovery rate for multiple comparisons [34]. An alpha level of 0.05 was set as the threshold for significance. All statistical analyses were performed in IBM SPSS Statistics 25.0.0.1.

The study was conducted according to the principles of the Declaration of Helsinki (64th WMA, 2013) and the general Data Protection Regulation.

## Results

A total of 312 children were included in this study. Of these children, 208 were HA users, and 104 CI users (see Table 1 for baseline characteristics). There were 173 (55%) boys, and 139 (45%) girls. Ages ranged from 4 to 18 years (mean ± standard deviation (M ± SD) = 10.5 ± 3.8). Children were diagnosed with hearing loss at a median age of 5 months (inter quartile range (IQR) = 1–52), and received hearing amplification at a median age of 32 months (IQR = 13–75). Children with CI were fitted with Cochlear^®^ (Nucleus 6, Nucleus 7, Kanso 1, Kanso 2) or Advanced Bionics^®^ (Naida Q70, Naida Q90, Sky) device(s). The children with HA used conventional HA(s; *n* = 192) or bone conducting device(s; *n* = 16) of various manufacturers. Hearing devices were worn bilaterally by 234 (75%) of the children. Twenty seven (9%) had bimodal hearing (CI + HA). Children had co-occurring conditions in 125 (40%) of the cases, such as Usher syndrome, physical or cerebral developmental delay, Muenke syndrome, cleft palate, and CHARGE syndrome.

**Table 1:** Demographics of the children that had an SDQ filled out prior to their appointment

Characteristics	Cochlear implant (*n* = 104)	Hearing aid (*n* = 208)	*p* value
Sex			0.38
Male	54 (52%)	119 (75%)	
Female	50 (48%)	89 (25%)	
Age, years			
Mean	11	10	0.09
Range	4–18	4–18	
Unaided pure-tone thresholds (PTA 0.5–4 kHz, better ear)			
*n*		201	
Mild	–	127 (63%)	n/a
Moderate	–	64 (32%)	
Severe	–	8 (4%)	
Profound	–	2 (1%)	
Type of hearing loss			
*n*		201	
Conductive	–	51 (25%)	n/a
Sensorineural	104 (100%)	150 (75%)	
Hearing device			
Hearing aid	–	192 (92%)	
Bone conducting device	–	16 (8%)	n/a
Age at diagnosis, months			
Median [IQR]	5 [2 to 29]	7 [1 to 64]	0.29
Age at device, months			
Median [IQR]	21 [15 to 39]	53 [8 to 86]	0.15
Side of amplification			0.10
Unilateral	13 (12%)	61 (29%)	
Bilateral	91 (88%)	147 (71%)	
Modality of amplification			< 0.01*
Unimodal (CI or HA)	89 (86%)	193 (93%)	
Bimodal (CI + HA)	15 (14%)	15 (7%)	
Presence of additional needs			< 0.01*
Yes	18 (17%)	90 (43%)	
No	86 (83%)	118 (57%)	
Speech perception in quiet (binaural, 45 dB SPL, %)			
*n*	76	147	
Median [IQR]	86 [77 to 92]	91 [79 to 97]	0.40
Speech perception in noise (binaural, SNR)			
*n*	59	53	
Median [IQR]	− 2.7 [− 4.0 to − 0.4]	− 6.1 [− 7.0 to − 3.9]	< 0.01*
Type of education			
Mainstream	52 (50%)	150 (72%)	< 0.01*
Special^a^	45 (43%)	18 (9%)	
Other^b^	7 (7%)	40 (19%)	

Demographics of the population are denoted per group of hearing device. Pure-tone thresholds were calculated as the pure-tone average on 0.5, 1, 2, and 4 kHz in the better hearing ear. Speech perception in quiet was measured with the NVA list, in aided condition. Speech perception in noise was measured with the digits in noise test in aided condition. A hyphen minus indicates that the characteristic was inapplicable to the group. Significance on the analysis of variance is indicated by bold print and asterisk. PTA is an abbreviation of pure-tone average; kHz, kilohertz; dB SPL, decibels sound pressure level; IQR, inter quartile range; SNR, signal to noise ratio; SD, standard deviation

^a^Special education for the deaf

^b^Other education comprises all other types of special schooling

Of the 450 primarily invited, 312 parents completed the form. The response rate was, therefore, 69%. Informants spent a median of 3 min to complete the SDQ, with 19 informants taking longer than 20 min.

### Distribution of parent-reported SDQ scores

As there were no significant differences in parent-reported SDQ scores between children with CI and HA, descriptive results are presented for the entire group.

**Fig. 3:**
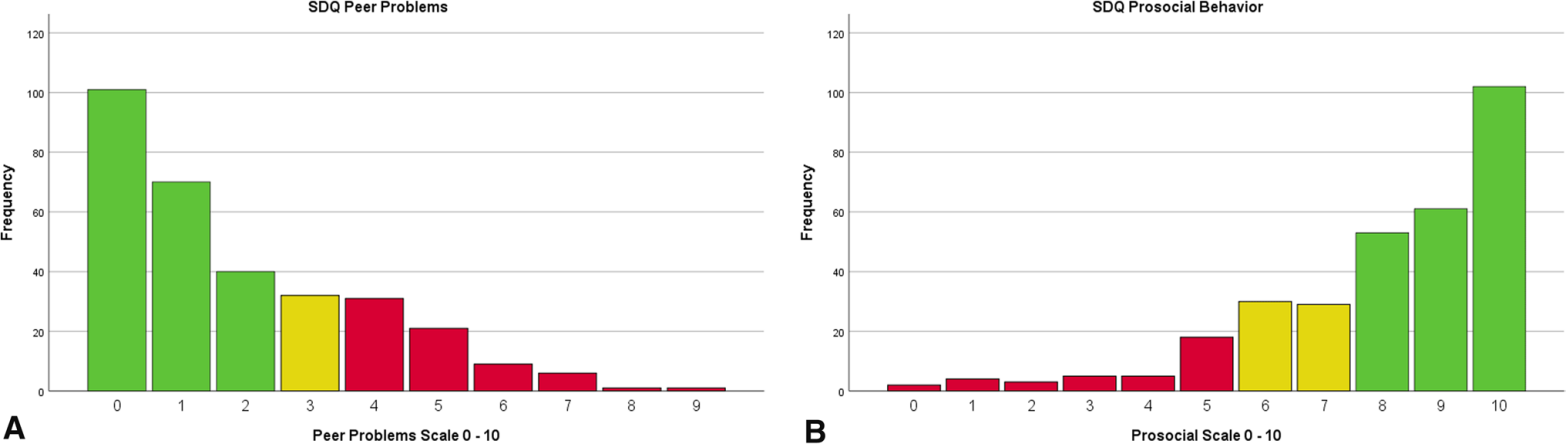
**A** Frequencies of difficulties measured in the peer problems domain. The histogram illustrates the distribution of parent-reported Peer problems. A green bar indicates scores close to average, yellow slightly raised, and red high and very high (i.e., clinical) scores. **B** Frequencies of difficulties measured in prosocial behavior. The histogram illustrates the distribution of parent-reported Prosocial behavior. A green bar indicates scores close to average, yellow slightly lowered, and red low and very low (i.e., clinical) scores. Scores should be interpreted from low (unfavorable) to high (favorable), since prosocial behavior is seen as strength in the SDQ

With regard to the first objective, we investigated the distribution of the SDQ scores. In the entire population, the median parent-reported SDQ Total difficulties score was 8 [IQR] = 4–12). The peer problems (median [IQR] = 1 [0–3]) and prosocial behavior (median [IQR] = 9 [7–10]) scales had a relatively large share in the elevated total difficulties scores. Ten percent of the children scored *high*, and another 12% scored *very high* in these categories. For the other scales, the scores (median [IQR]) were: emotional problems 1 [0–3], conduct problems 1 [0–2], hyperactivity 3 [1–5]. Table 2 shows the distribution of parent-reported SDQ scores. Histograms of the peer problems and prosocial behavior scores are provided in Fig. 3.

**Table 2:** Distribution of the parent-reported SDQ scores

SDQ Scale	Slightly raised			High			Very high		
	*n*	%	% of the normal population	*n*	%	% of the normal population	*n*	%	% of the normal population
Emotional	17	5	10	36	12	5	11	4	5
Conduct	27	9	10	24	8	5	4	2	5
Hyperactivity	39	13	10	12	4	5	15	5	5
Peer problems	32	10	10	31	10	5	38	12	5
Prosocial behavior	29	9	10	30	10	5	37	12	5
Total difficulties	14	4	10	19	6	5	18	6	5

Prevalence in the population is denoted behind every SDQ scale. In addition, the prevalence in the normal hearing population is provided for the bands *slightly raised, high,* and *very high*. Elevated prevalences are highlighted in blue. In case of prosocial behavior, the three bands should be interpreted as *slightly lowered, low,* and *very low,* respectively

Parent-reported total problems were in the clinical range in 37 (12%) of all 312 children, and 22% of all children had one or more problems in the clinical range.

Of the individual scales, peer problems and prosocial behavior problems were often clinically raised (22% for both scales). This prevalence was significantly higher than in a normal hearing children with similar age (i.e., 9%, *p* < 0.01; Maurice-Stam et al. 2018). In our sample, the prevalence of clinical peer problems was seen to be higher in 12–18 year olds (29%) compared to 6–11 year olds (19%; *p* < 0.05). A full account of the clinical parent-reported SDQ scores can be found in Table 3. Boys scored significantly higher on hyperactivity (4 [2–6] vs 3 [1–5], *p* = 0.01). No other differences were found between boys and girls.

**Table 3:** Distribution of clinical scores on the parent-reported SDQ stratified by age category

Characteristic	4–5 years			6–11 years			12–18 years		
	(*n* = 44)	Norm (%)	*p* value	(*n* = 155)	Norm (%)	*p* value	(*n* = 113)	Norm (%)	*p* value
SDQ scale									
Emotional	5 (11%)	8	0.51	23 (15%)	7	< 0.01*	19 (17%)	11	0.11
Conduct	7 (16%)	–		14 (9%)	7	0.47	7 (6%)	13	< 0.05*
Hyperactivity	5 (11%)	13	0.82	15 (10%)	12	0.46	7 (6%)	12	0.07
Peer problems	12 (27%)	–		24 (16%)^a^	12	0.23	33 (29%)^a^	9	< 0.01*
Prosocial behavior	17 (39%)^bc^	9	< 0.01*	29 (19%)^b^	9	< 0.01*	21 (19%)^c^	12	0.06
Total	6 (14%)	9	0.40	18 (12%)	10	0.62	13 (12%)	9	< 0.01*
At least one deviant score	13 (30%)			30 (19%)			26 (23%)		

Differences in clinical prevalence between age bands are indicated with superscript. In each row, figures marked with ^a^, ^b^, or ^c^ are significantly different. All figures unmarked are not significantly different

**Fig. 4:**
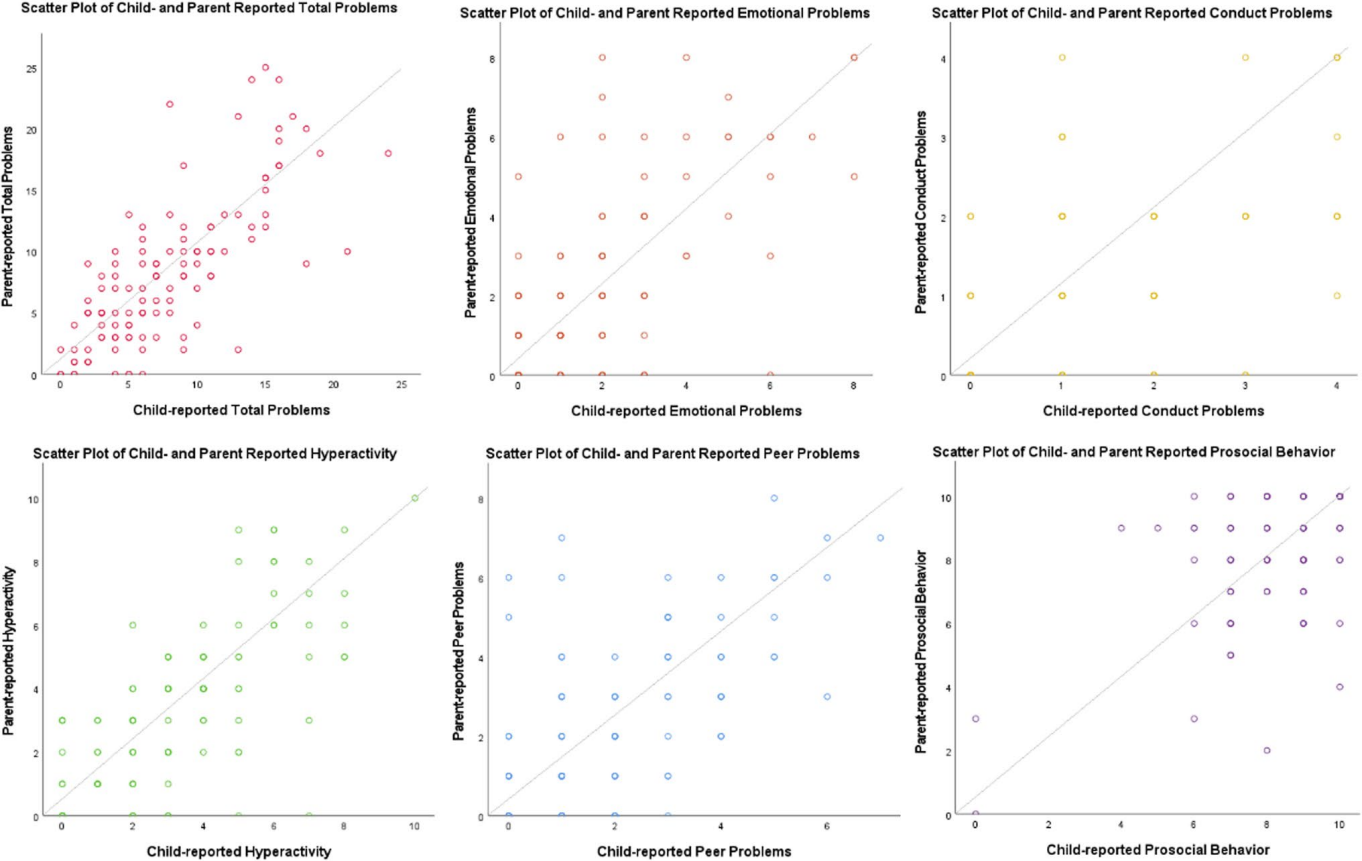
Scatter plots of child- and parent-reported difficulties on the same domains. These scatter plots illustrate the correlation between parent-reported and child-reported SDQ scores for the 113 children that had both parent- and child-reports available. *Y* = *X* diagonal is provided for reference. Dots near the diagonal represent parent-reported and child-reported scores with a high correlation. Dots plotted below the diagonal indicate a higher SDQ score reported by the child compared to the parent. Dots plotted above the diagonal indicate a higher parent-reported SDQ score compared to the child

Scores on all scales correlated significantly between parent- and child-reports. Of each scale, scatter plots are provided in Fig. 4.

### SDQ scores relative to hearing device, type of hearing loss, auditory performance, and education

With regard to the second objective, we investigated several auditory and educational factors expected to be related to SDQ scores.

Hearing thresholds were available of 201 of the HA users. No correlation between all scales on the parent- and child-reported SDQs and the children’s PTA4 was found. No difference was observed in parent-rated SDQ scores between children with conductive- or perceptive HL. We found no difference in scores between children with CIs and children with HAs.

Poorer speech perception in quiet was associated with more parent-reported conduct problems (*r*_s_ = − 0.18, *p* < 0.01, *N* = 223). Other scales were not significantly associated with speech perception in quiet.

Speech perception in noise scores were available of 112 children. CI users had a significantly higher signal to noise ratio (SNR) than HA users (− 1.2; vs. − 5.3, *p* < 0.01). No association was found between all scales on the parent-reported SDQ and the children’s speech perception in noise.

Children received *mainstream* education in 65% (*n* = 202) of the cases, 20% (*n* = 63) received *special* education for the deaf, and 15% (*n* = 47) received any other type of special schooling (i.e., *other*). *Other* schooling comprised of daycare or primary/secondary education for children with multiple disabilities (*n* = 28), education for children with severe learning difficulties (*n* = 10), and special primary/secondary education (*n* = 9).

### Hearing aid users’ parent-reported SDQ scores with respect to education and additional needs

**Fig. 5:**
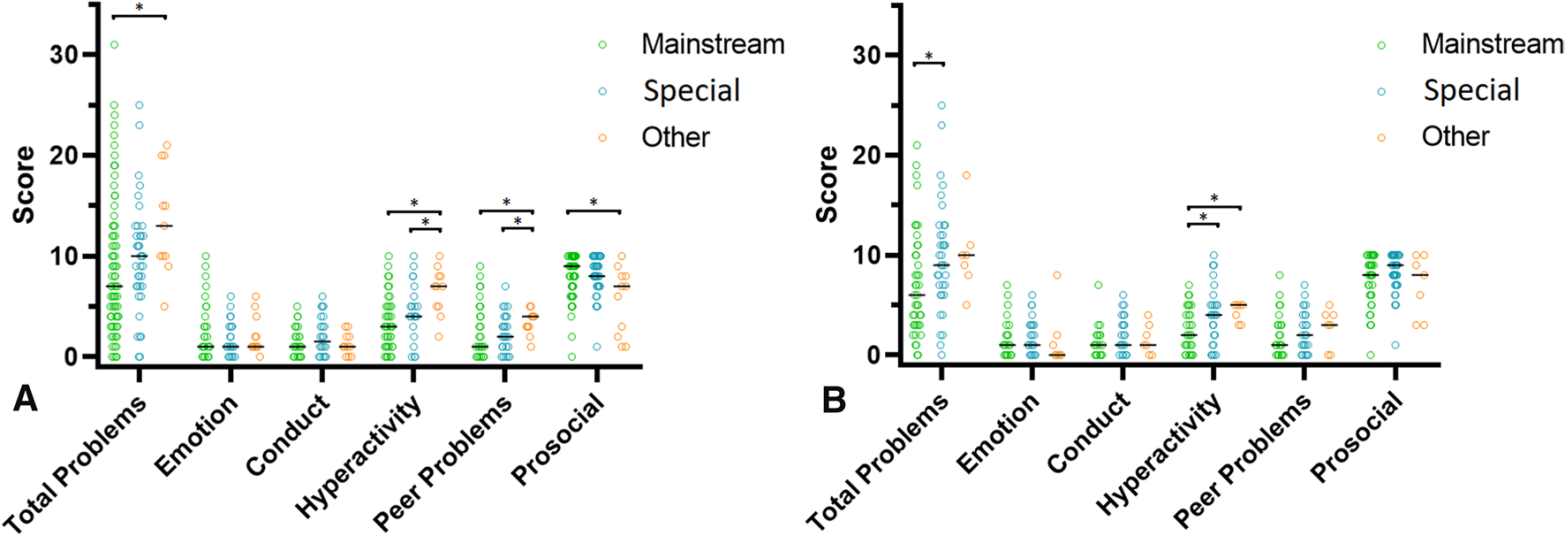
**A** HA users’ parent-reported SDQ scores by education. Distribution per SDQ scale is provided per education type for HA users. The mean value for each group is indicated by the black dashes. Higher scores indicate more problems, except for the prosocial behavior scale, in which higher scores indicate more favorable behavior. Significant differences are illustrated by an asterisk. **B** CI users’ parent-reported SDQ scores by education. The distribution per SDQ scale is provided per education type for CI users. The mean value for each group is indicated by the black dashes. Higher scores indicate more problems, except for the prosocial behavior scale, in which higher scores indicate more favorable behavior. Significant differences are illustrated by an asterisk

Among HA users, parent-reported SDQ scores of children in *special* education were similar to those in *mainstream* education (Fig. 5A), whereas children in *other* education scored higher on total difficulties (median [IQR]: *mainstream* = 7 [3.8–11]; vs. *other* = 12 [9–16.5], *p* < 0.01), hyperactivity (median, IQR: *mainstream* = 3 [1–5]; vs. *other* = 6 [3.3–8], *p* < 0.01), peer problems (median [IQR]: *mainstream* = 1 [0–2]; vs. *other* = 3.5 [1–4], *p* < 0.01), and lower on prosocial behavior (median [IQR]: *mainstream* = 9 [8–10]; vs. *other* = 7.5 [6–8.8], *p* < 0.01). Scores in other scales were not significantly different across education groups.

Additional needs were present in 90 (43%) of the 208 children with HA. A Mann–Whitney test revealed significantly higher parent-reported SDQ scores in most scales for children with additional needs compared to those without. Total difficulties median = 10 and 6 respectively, *U* = 7310, *p* < 0.01; conduct problems median = 1 and 1, respectively, *U* = 6591, *p* < 0.01; hyperactivity median = 4 and 3, respectively, *U* = 6951, *p* < 0.01; peer problems median = 2 and 1, respectively, *U* = 7481, *p* < 0.01; prosocial scale median = 8 and 9, respectively, *U* = 3704, *p* < 0.01.

### Cochlear implant users’ parent-reported SDQ scores with respect to education and additional needs

For CI users, the parent-reported total difficulties were significantly higher in children following *special* education (median [IQR]: *mainstream* = 5.5 [3–10]; vs. *special* = 9 [6–12.5], *p* = 0.02). This difference was mainly due to the elevated hyperactivity scores in *special* education (median [IQR]: *mainstream* = 2 [1–4]; vs. *special* = 4 [1–5], *p* = 0.04). See Fig. 5B for the distribution of parent-reported SDQ scores.

Additional needs were present in 18 (17%) of the 104 children with CI. No differences between children with or without additional needs were present in parent- and child-reported SDQ total scores and subscales.

## Discussion

We implemented the SDQ in the children’s clinical follow-up in the audiology department of Erasmus University and Medical Center in Rotterdam. This allowed us to identify and address any psychosocial challenges that some of the children in our clinical group may be experiencing.

In almost one in four of the children with reduced hearing we found clinically relevant problems with peer relationships and prosocial behavior. Either one or both of these two scales were consistently elevated across age groups. This suggests that children with hearing loss (HL) are at a constant risk experiencing constraints in interaction (peer problems), and relationships in social contexts (prosocial behavior). Through the years, similar findings have been reported by several studies both using the SDQ and other investigative tools. Stevenson et al., summarized that among children with reduced hearing, peer problems are most common. A feasible explanation is that HL impairs communication and social interactions. Isolation from social interactions may result in lower self-esteem, and trust in social acceptance [35, 36]. Another factor that may contribute to psychosocial difficulties in children with hearing loss is having additional needs. When children with hearing loss have additional needs, such as learning or health-related needs, it can create obstacles for them to navigate daily life and interact with others. Therefore, these difficulties may stem not only from their hearing loss but also from their additional needs. It is, therefore, important to consider the potential impact of these needs when assessing and providing support for children with hearing loss.

Various protective factors against social isolation and psychosocial difficulties have been presented. Several research groups investigated auditory functioning in terms of speech perception in association with psychosocial difficulties. Better speech perception, both in quiet and in more challenging listening environments, is associated with better psychosocial outcomes [8, 12, 23, 24]. In our study, we found that children with better speech perception in quiet were less likely to evince behavioral issues (conduct). Although this was a modest correlation, it substantiates the hypothesis that children’s mental health may depend on their communication possibilities. School environments may play a substantial part in the development and integration of communication abilities, and in turn, children’s psychosocial wellbeing. Therefore, we investigated the distribution of psychosocial difficulties across different types of education. In our study, children showed similar psychosocial outcomes between mainstream education and special education for the deaf, apart from hyperactivity, which may be due to the increased prevalence of additional needs in children in special schooling.

Despite these findings, there are still limitations to our understanding of the complex relationship between auditory functioning, type of education, and psychosocial difficulties. Therefore, it is important to continue investigating these factors in order to better support the psychosocial wellbeing of these children.

This is the first study to investigate the SDQ as a screening tool during the clinical follow-up of children with HL. It is also one of the largest recent studies that investigated the prevalence and severity of psychosocial difficulties in these children. In addition, the findings on auditory functioning and type of education are a valuable addition to the general knowledge of the intricate framework of HL, (social) interaction, and psychosocial health. Several limitations to this study do exist: (1) the cross-sectional design did not allow for the investigation of causality between the identified associations; (2) the findings could be influenced by response bias; (3) not all children had auditory scores available, therefore, sampling bias could not be ruled out completely; (4) SDQ results were obtained during the COVID19 pandemic, which may have influenced the children’s psychosocial wellbeing. Lastly, we could not perform a structured evaluation of the clinical consequences that screening had. Future research should investigate the effect of screening on psychosocial difficulties and referral and possible psychological assessment and intervention.

## Conclusions

The present study implemented the Strengths and Difficulties Questionnaire (SDQ) in the clinical follow-up of children with HL in order to assess psychosocial difficulties and identify potential predictors. The results showed that almost one in four of the children had clinically relevant problems with peer relationships and prosocial behavior, which were consistently elevated across age groups. These findings suggest that children with HL are at a constant risk of experiencing constraints in social interactions and attachment in social contexts. Better speech perception abilities were found to be associated with fewer behavioral issues, supporting the hypothesis that children's mental health may depend on their communication abilities. Also psychosocial outcomes in mainstream education and special education for the deaf were mostly similar. The clinical implementation of the SDQ may increase the chances for early psychological assessment and intervention.

## Supplementary Information

Below is the link to the electronic supplementary material.

Supplementary file1 (DOCX 131 KB)

## Data Availability

The data that support the findings of this study are available on request from the corresponding author, TJJ. The data are not publicly available due to their containing information that could compromise the privacy of research participants.
