# Supplementary material for: First results of the Strengths and Difficulties Questionnaire, applied as a screening tool for psychosocial difficulties in pediatric audiology
Source: Eur Arch Otorhinolaryngol. 2023 Apr 21;280(10):4467–76. doi: 10.1007/s00405-023-07979-x (PMC10477219; doi:10.1007/s00405-023-07979-x)
Supplement: Supplementary file 1 — Supplementary file1 (DOCX 131 KB) [file 405_2023_7979_MOESM1_ESM.docx]

| 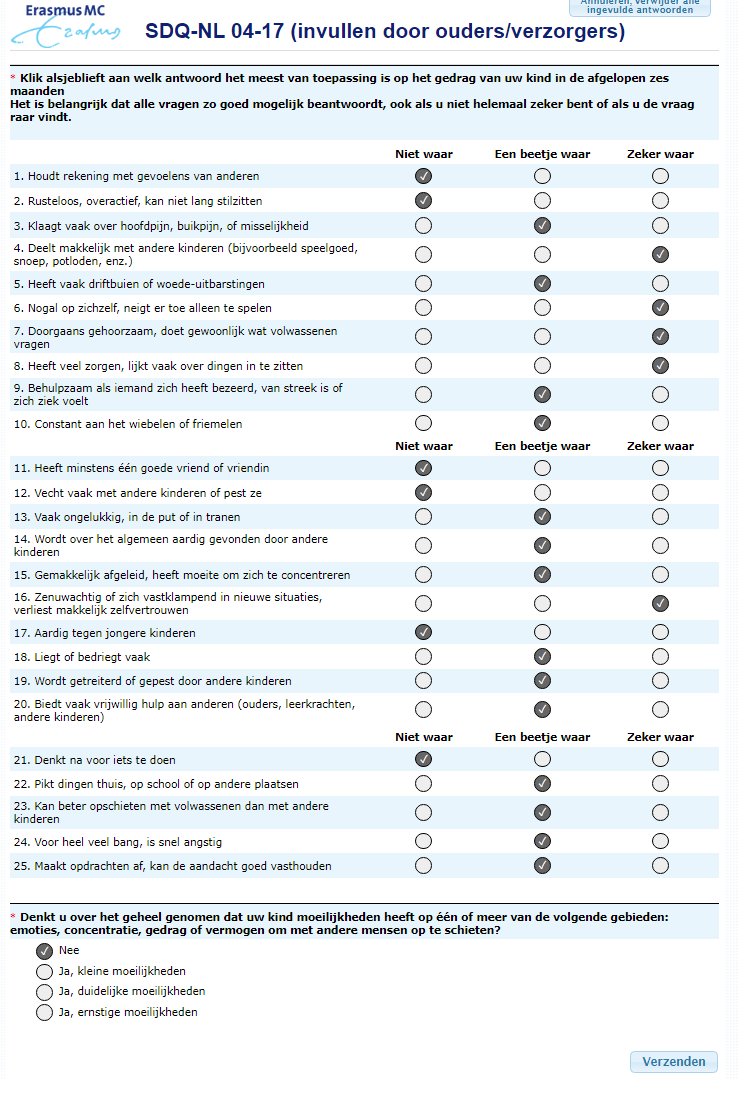 |
| --- |
| **Supplementary Figure 1. The online version of the Dutch Strengths and Difficulties Questionnaire.** The finches are inserted as example. |
